# *rac*-2-Amino­pyridinium *cis*-2-carb­oxy­cyclo­hexane-1-carboxyl­ate

**DOI:** 10.1107/S1600536811025256

**Published:** 2011-07-02

**Authors:** Graham Smith, Urs D. Wermuth

**Affiliations:** a Faculty of Science and Technology, Queensland University of Technology, GPO Box 2434, Brisbane, Queensland 4001, Australia

## Abstract

In the structure of the title compound, C_5_H_7_N_2_^+^·C_8_H_11_O_4_^−^, the *cis* anions associate through head-to-tail carb­oxy­lic acid–carboxyl O—H⋯O hydrogen bonds [graph set *C*(7)], forming chains which extend along *c* and are inter­linked through the carboxyl groups, forming cyclic *R*_2_^2^(8) associations with the pyridinium and an amine H-atom donor of the cation. Further amine–carboxyl N—H⋯O inter­actions form enlarged centrosymmetric rings [graph set *R*_4_^4^(18)] and extensions down *b*, giving a three-dimensional structure.

## Related literature

For the structure of racemic *cis*-cyclo­hexane,1,2-dicarb­oxy­lic acid, see: Benedetti *et al.* (1970 ▶). For the structure of racemic ammonium *cis*-2-carb­oxy­cyclo­hexane-1-carboxyl­ate, see: Smith & Wermuth (2011 ▶) and of brucinium (1*R*,2*S*-2-carb­oxy­cyclo­hexane-1-carboxyl­ate dihydrate, see: Smith *et al.* (2011 ▶). For the structure of the adduct of *cis*-cyclo­hexane-1,2-dicarb­oxy­lic acid with 4,4′-bipyridine, see: Bhogala *et al.* (2005 ▶). For graph-set analysis, see: Etter *et al.* (1990) ▶.
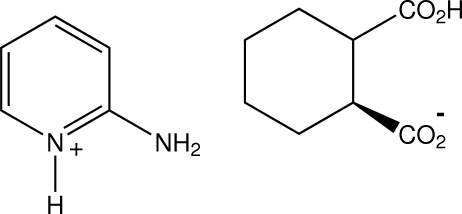


## Experimental

### 

#### Crystal data

C_5_H_7_N_2_^+^·C_8_H_11_O_4_^−^
*M* *_r_* = 266.29
Monoclinic, 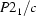

*a* = 12.4709 (5) Å
*b* = 10.4191 (5) Å
*c* = 10.6451 (5) Å
β = 101.250 (4)°
*V* = 1356.60 (11) Å^3^
*Z* = 4
Mo *K*α radiation
μ = 0.10 mm^−1^
*T* = 200 K
0.35 × 0.32 × 0.20 mm

#### Data collection

Oxford Diffraction Gemini-S CCD-detector diffractometer
Absorption correction: multi-scan (*CrysAlis PRO*; Oxford Diffraction, 2010 ▶) *T*_min_ = 0.90, *T*_max_ = 0.98
9200 measured reflections
2658 independent reflections
1947 reflections with *I* > 2σ(*I*)
*R*_int_ = 0.022

#### Refinement

*R*[*F* ^2^ > 2σ(*F* ^2^)] = 0.033
*wR*(*F* ^2^) = 0.081
*S* = 0.99
2658 reflections
188 parameters
H atoms treated by a mixture of independent and constrained refinement
Δρ_max_ = 0.16 e Å^−3^
Δρ_min_ = −0.15 e Å^−3^

### 

Data collection: *CrysAlis PRO* (Oxford Diffraction, 2010 ▶); cell refinement: *CrysAlis PRO*; data reduction: *CrysAlis PRO*; program(s) used to solve structure: *SIR92* (Altomare *et al.*, 1994 ▶); program(s) used to refine structure: *SHELXL97* (Sheldrick, 2008 ▶) within *WinGX* (Farrugia, 1999 ▶); molecular graphics: *PLATON* (Spek, 2009 ▶); software used to prepare material for publication: *PLATON*.

## Supplementary Material

Crystal structure: contains datablock(s) global, I. DOI: 10.1107/S1600536811025256/wn2440sup1.cif

Structure factors: contains datablock(s) I. DOI: 10.1107/S1600536811025256/wn2440Isup2.hkl

Additional supplementary materials:  crystallographic information; 3D view; checkCIF report

## Figures and Tables

**Table 1:** Hydrogen-bond geometry (Å, °)

*D*—H⋯*A*	*D*—H	H⋯*A*	*D*⋯*A*	*D*—H⋯*A*
N1*A*—H1*A*⋯O11	0.981 (15)	1.656 (15)	2.6223 (15)	167.7 (14)
N21*A*—H21*A*⋯O12	0.911 (18)	2.044 (17)	2.9361 (16)	166.3 (14)
N21*A*—H22*A*⋯O22^i^	0.873 (16)	2.105 (16)	2.9103 (17)	153.1 (14)
O21—H21⋯O11^ii^	0.991 (19)	1.595 (19)	2.5806 (13)	172.9 (18)

Symmetry codes: (i) 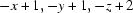; (ii) 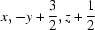.

**Fig. 1.**
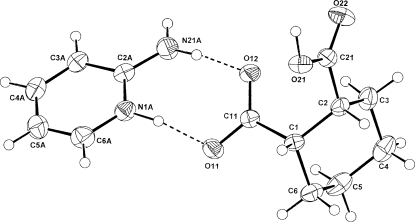
Molecular configuration for the cation and anion species. Inter-species hydrogen bonds are shown as dashed lines and displacement ellipsoids are drawn at the 40% probability level.

**Fig. 2.**
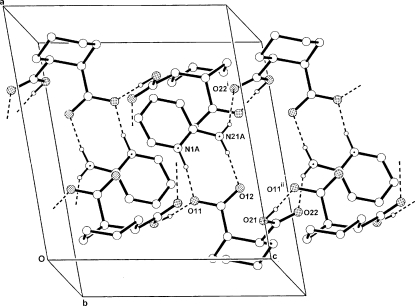
The inter-associated duplex cis-CHDC anion chains which extend along the c direction in the unit cell, showing hydrogen-bonding interactions as dashed lines. Non-associative H atoms are omitted. For symmetry codes, see Table 1.

**Fig. 3.**
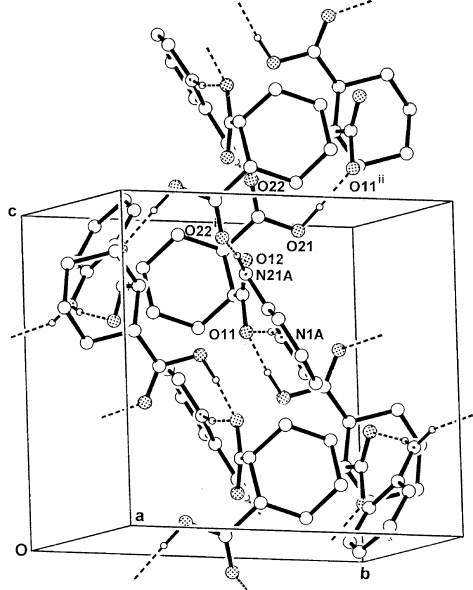
A view of the hydrogen-bonding extensions in the structure down the b axis.

## supplementary crystallographic information

### Comment

Although the structure of racemic *cis*-cyclohexane-1,2-dicarboxylic acid (*cis*-CHDC) is known (Benedetti *et al.*, 1970), together with its 1:1 adduct with 4,4'-bipyridine (Bhogala *et al.*, 2005), there are few examples of salts of this acid in the crystallographic literature. We have now reported the structures of the 1:1 ammonium salt (Smith & Wermuth, 2011) and the 1:1 brucinium salt trihydrate (Smith *et al.*, 2011), in which the 1*R*,2*S* enantiomer of *cis*-CHDC has been resolved. Our 1:1 stoichiometric reaction of cyclohexane-1,2-dicarboxylic anhydride with 2-aminopyridine in 50% ethanol/water solution gave large crystals of the title compound, C_5_H_7_N_2_^+^ C_8_H_11_O_4_^-^  and the structure is reported here.

In the structure (Fig. 1) of the title compound, *cis*C_5_H_7_N_2_^+^ C_8_H_11_O_4_^-^, the *cis*-anions associate through head-to-tail carboxylic acid–carboxyl O—H···O hydrogen-bonds [graph set C(7) (Etter *et al.*, 1990)] and are inter-linked  through the carboxyl groups, forming cyclic R^2^_2_(8) associations with the pyridinium and an amine H donor of the cation. Further amine···carboxyl N—H···O interactions (Table 1) form enlarged centrosymmetric rings [graph set R^4^_4_(18)] (Fig. 2) and extensions down *b* (Fig. 3) to give a three-dimensional structure.

### Experimental

The title compound was synthesized by heating a solution of 1 mmol of cyclohexane-1,2-dicarboxylic anhydride and 1 mmol of 2-aminopyridine in 50 ml of 1:1 ethanol–water under reflux for 10 min. After concentration to 30 ml the solution was allowed evaporate to moist dryness at room temperature, giving large colourless plates of the title compound (m.p. 396 K) from which a specimen was cleaved for the X-ray analysis.

### Refinement

Hydrogen atoms involved in hydrogen-bonding interactions were located by difference methods and their positional and isotropic displacement parameters were refined. Other H-atoms were included in the refinement at calculated positions [C—H = 0.93–0.98 Å and with *U*_iso_(H) = 1.2*U*_eq_(C), using a riding-model approximation.

### Crystal data

**Table:**

C_5_H_7_N_2_^+^·C_8_H_11_O_4_^−^	*F*(000) = 568
*M**_r_* = 266.29	*D*_x_ = 1.304 Mg m^−^^3^
Monoclinic, *P*2_1_/*c*	Melting point: 396 K
Hall symbol: -P 2ybc	Mo *K*α radiation, λ = 0.71073 Å
*a* = 12.4709 (5) Å	Cell parameters from 4347 reflections
*b* = 10.4191 (5) Å	θ = 3.4–28.7°
*c* = 10.6451 (5) Å	µ = 0.10 mm^−^^1^
β = 101.250 (4)°	*T* = 200 K
*V* = 1356.60 (11) Å^3^	Block, colourless
*Z* = 4	0.35 × 0.32 × 0.20 mm

### Data collection

**Table:**

Oxford Diffraction Gemini-S CCD-detector diffractometer	2658 independent reflections
Radiation source: Enhance (Mo) X-ray source	1947 reflections with *I* > 2σ(*I*)
graphite	*R*_int_ = 0.022
Detector resolution: 16.077 pixels mm^-1^	θ_max_ = 26.0°, θ_min_ = 3.4°
ω scans	*h* = −15→15
Absorption correction: multi-scan (*CrysAlis PRO*; Oxford Diffraction, 2010)	*k* = −12→12
*T*_min_ = 0.90, *T*_max_ = 0.98	*l* = −13→13
9200 measured reflections	

### Refinement

**Table:**

Refinement on *F*^2^	Primary atom site location: structure-invariant direct methods
Least-squares matrix: full	Secondary atom site location: difference Fourier map
*R*[*F*^2^ > 2σ(*F*^2^)] = 0.033	Hydrogen site location: inferred from neighbouring sites
*wR*(*F*^2^) = 0.081	H atoms treated by a mixture of independent and constrained refinement
*S* = 0.99	*w* = 1/[σ^2^(*F*_o_^2^) + (0.0472*P*)^2^] where *P* = (*F*_o_^2^ + 2*F*_c_^2^)/3
2658 reflections	(Δ/σ)_max_ = 0.001
188 parameters	Δρ_max_ = 0.16 e Å^−^^3^
0 restraints	Δρ_min_ = −0.15 e Å^−^^3^

### Special details

**Table:**

Geometry. Bond distances, angles *etc*. have been calculated using the rounded fractional coordinates. All su's are estimated from the variances of the (full) variance-covariance matrix. The cell e.s.d.'s are taken into account in the estimation of distances, angles and torsion angles
Refinement. Refinement of *F*^2^ against ALL reflections. The weighted *R*-factor *wR* and goodness of fit *S* are based on *F*^2^, conventional *R*-factors *R* are based on *F*, with *F* set to zero for negative *F*^2^. The threshold expression of *F*^2^ > σ(*F*^2^) is used only for calculating *R*-factors(gt) *etc*. and is not relevant to the choice of reflections for refinement. *R*-factors based on *F*^2^ are statistically about twice as large as those based on *F*, and *R*- factors based on ALL data will be even larger.

### Fractional atomic coordinates and isotropic or equivalent isotropic displacement parameters (Å^2^)

**Table:**

	*x*	*y*	*z*	*U*_iso_*/*U*_eq_	
O11	0.29045 (7)	0.58197 (9)	0.65073 (8)	0.0412 (3)	
O12	0.34131 (6)	0.57102 (9)	0.86187 (8)	0.0405 (3)	
O21	0.18216 (7)	0.77924 (9)	0.96839 (9)	0.0411 (3)	
O22	0.23826 (8)	0.62543 (10)	1.11054 (8)	0.0494 (3)	
C1	0.14901 (9)	0.59059 (12)	0.77138 (11)	0.0310 (4)	
C2	0.12817 (9)	0.56727 (12)	0.90706 (12)	0.0327 (4)	
C3	0.14384 (10)	0.42684 (13)	0.94720 (14)	0.0426 (4)	
C4	0.07163 (11)	0.34055 (14)	0.85100 (16)	0.0529 (5)	
C5	0.09356 (11)	0.36091 (14)	0.71709 (16)	0.0521 (5)	
C6	0.07708 (10)	0.50055 (13)	0.67677 (13)	0.0422 (4)	
C11	0.26966 (10)	0.57955 (11)	0.76400 (12)	0.0308 (4)	
C21	0.19003 (9)	0.65774 (13)	1.00503 (12)	0.0341 (4)	
N1A	0.49126 (9)	0.62635 (11)	0.62208 (11)	0.0387 (4)	
N21A	0.55457 (11)	0.50551 (13)	0.80144 (12)	0.0474 (5)	
C2A	0.57435 (10)	0.56346 (12)	0.69688 (13)	0.0356 (4)	
C3A	0.67632 (10)	0.56253 (13)	0.65925 (13)	0.0410 (4)	
C4A	0.68805 (11)	0.62558 (14)	0.55114 (14)	0.0467 (5)	
C5A	0.60091 (12)	0.69282 (14)	0.47783 (14)	0.0509 (5)	
C6A	0.50392 (12)	0.69068 (14)	0.51527 (14)	0.0473 (5)	
H1	0.12600	0.67860	0.74740	0.0370*	
H2	0.05060	0.58600	0.90310	0.0390*	
H21	0.2185 (15)	0.8363 (19)	1.0383 (18)	0.089 (6)*	
H31	0.21980	0.40290	0.95300	0.0510*	
H32	0.12570	0.41550	1.03110	0.0510*	
H41	0.08540	0.25150	0.87570	0.0640*	
H42	−0.00460	0.35890	0.85140	0.0640*	
H51	0.04460	0.30730	0.65710	0.0630*	
H52	0.16800	0.33550	0.71500	0.0630*	
H61	0.00090	0.52350	0.67120	0.0510*	
H62	0.09440	0.51130	0.59240	0.0510*	
H1A	0.4183 (12)	0.6177 (14)	0.6427 (14)	0.055 (4)*	
H3A	0.73520	0.51910	0.70800	0.0490*	
H4A	0.75520	0.62400	0.52550	0.0560*	
H5A	0.60970	0.73790	0.40510	0.0610*	
H6A	0.44470	0.73400	0.46710	0.0570*	
H21A	0.4889 (14)	0.5126 (15)	0.8261 (15)	0.060 (5)*	
H22A	0.6077 (13)	0.4646 (15)	0.8509 (15)	0.054 (4)*	

### Atomic displacement parameters (Å^2^)

**Table:**

	*U*^11^	*U*^22^	*U*^33^	*U*^12^	*U*^13^	*U*^23^
O11	0.0347 (5)	0.0558 (6)	0.0334 (5)	0.0086 (4)	0.0076 (4)	0.0050 (5)
O12	0.0271 (4)	0.0577 (6)	0.0347 (5)	0.0022 (4)	0.0011 (4)	0.0019 (4)
O21	0.0456 (5)	0.0376 (5)	0.0356 (5)	0.0017 (4)	−0.0031 (4)	−0.0025 (4)
O22	0.0553 (6)	0.0572 (7)	0.0314 (5)	0.0134 (5)	−0.0024 (4)	0.0036 (5)
C1	0.0273 (6)	0.0309 (7)	0.0323 (7)	0.0041 (5)	−0.0002 (5)	0.0005 (5)
C2	0.0222 (6)	0.0373 (7)	0.0380 (7)	0.0045 (5)	0.0044 (5)	0.0027 (6)
C3	0.0362 (7)	0.0406 (8)	0.0515 (8)	0.0003 (6)	0.0101 (6)	0.0107 (7)
C4	0.0414 (8)	0.0384 (8)	0.0781 (11)	−0.0057 (7)	0.0094 (7)	0.0034 (8)
C5	0.0402 (8)	0.0424 (9)	0.0703 (10)	−0.0070 (6)	0.0023 (7)	−0.0179 (8)
C6	0.0309 (6)	0.0505 (9)	0.0418 (8)	−0.0017 (6)	−0.0009 (6)	−0.0083 (7)
C11	0.0307 (6)	0.0280 (7)	0.0327 (7)	0.0026 (5)	0.0034 (5)	0.0007 (5)
C21	0.0262 (6)	0.0429 (8)	0.0332 (7)	0.0082 (5)	0.0061 (5)	0.0022 (6)
N1A	0.0327 (6)	0.0385 (6)	0.0435 (7)	0.0042 (5)	0.0040 (5)	0.0024 (5)
N21A	0.0344 (7)	0.0615 (9)	0.0462 (8)	0.0105 (6)	0.0073 (6)	0.0133 (6)
C2A	0.0319 (6)	0.0336 (7)	0.0392 (7)	0.0008 (5)	0.0018 (6)	−0.0030 (6)
C3A	0.0311 (7)	0.0437 (8)	0.0468 (8)	0.0005 (6)	0.0039 (6)	−0.0004 (7)
C4A	0.0391 (8)	0.0464 (9)	0.0558 (9)	−0.0082 (6)	0.0121 (7)	−0.0023 (7)
C5A	0.0539 (9)	0.0479 (9)	0.0511 (9)	−0.0059 (7)	0.0109 (7)	0.0115 (7)
C6A	0.0473 (8)	0.0414 (8)	0.0498 (9)	0.0045 (7)	0.0009 (7)	0.0083 (7)

### Geometric parameters (Å, °)

**Table:**

O11—C11	1.2821 (15)	C1—H1	0.9800
O12—C11	1.2363 (15)	C2—H2	0.9800
O21—C21	1.3226 (16)	C3—H32	0.9700
O22—C21	1.2137 (15)	C3—H31	0.9700
O21—H21	0.991 (19)	C4—H42	0.9700
N1A—C6A	1.3554 (19)	C4—H41	0.9700
N1A—C2A	1.3473 (17)	C5—H51	0.9700
N21A—C2A	1.3310 (19)	C5—H52	0.9700
N1A—H1A	0.981 (15)	C6—H61	0.9700
N21A—H21A	0.911 (18)	C6—H62	0.9700
N21A—H22A	0.873 (16)	C2A—C3A	1.4059 (18)
C1—C2	1.5359 (17)	C3A—C4A	1.358 (2)
C1—C11	1.5262 (17)	C4A—C5A	1.396 (2)
C1—C6	1.5319 (18)	C5A—C6A	1.346 (2)
C2—C3	1.5261 (19)	C3A—H3A	0.9300
C2—C21	1.5025 (18)	C4A—H4A	0.9300
C3—C4	1.519 (2)	C5A—H5A	0.9300
C4—C5	1.518 (2)	C6A—H6A	0.9300
C5—C6	1.520 (2)		
			
O11···N1A	2.6223 (15)	C11···H21^i^	2.520 (19)
O11···C21^i^	3.2513 (16)	C11···H52	2.8400
O11···O21^i^	2.5806 (13)	C11···H31	2.8800
O11···C4A^ii^	3.0969 (17)	C21···H42^viii^	3.0200
O11···O22^i^	3.1292 (14)	C21···H22A^iv^	2.972 (16)
O12···C6A^iii^	3.4134 (17)	H1···O21	2.5500
O12···C3	3.1653 (16)	H1···C4^v^	3.0000
O12···O22	3.2105 (12)	H1···H42^v^	2.5100
O12···N21A	2.9361 (16)	H1A···C11	2.489 (15)
O12···C21	2.7964 (15)	H1A···O11	1.656 (15)
O21···C11	3.3436 (15)	H1A···O12	2.734 (15)
O21···O11^iii^	2.5806 (13)	H1A···H21^i^	2.57 (2)
O22···O12	3.2105 (12)	H1A···H21A	2.26 (2)
O22···C3A^iv^	3.1555 (16)	H2···H61	2.5100
O22···O11^iii^	3.1292 (14)	H2···H32^viii^	2.4300
O22···N21A^iv^	2.9103 (17)	H2···H42	2.5000
O22···C2A^iv^	3.4158 (16)	H3A···H22A	2.4700
O11···H62	2.5100	H3A···O22^iv^	2.4200
O11···H4A^ii^	2.8300	H4A···H41^vii^	2.4500
O11···H21A	2.886 (17)	H4A···O11^ii^	2.8300
O11···H1A	1.656 (15)	H5A···C2A^i^	3.0000
O11···H21^i^	1.595 (19)	H5A···N21A^i^	2.9200
O12···H31	2.6200	H6A···O12^i^	2.5500
O12···H21A	2.044 (17)	H21···O11^iii^	1.595 (19)
O12···H1A	2.734 (15)	H21···C1^iii^	2.886 (19)
O12···H6A^iii^	2.5500	H21···H1A^iii^	2.57 (2)
O21···H62^iii^	2.8800	H21···H62^iii^	2.3700
O21···H1	2.5500	H21···C6^iii^	3.031 (19)
O21···H51^v^	2.9000	H21···C11^iii^	2.520 (19)
O22···H32	2.6500	H21A···C11	2.774 (18)
O22···H3A^iv^	2.4200	H21A···O11	2.886 (17)
O22···H22A^iv^	2.105 (16)	H21A···H1A	2.26 (2)
O22···H31	2.8400	H21A···O12	2.044 (17)
N1A···O11	2.6223 (15)	H22A···H3A	2.4700
N1A···C11	3.4331 (17)	H22A···O22^iv^	2.105 (16)
N21A···O12	2.9361 (16)	H22A···C21^iv^	2.972 (16)
N21A···O22^iv^	2.9103 (17)	H31···O12	2.6200
N21A···H5A^iii^	2.9200	H31···O22	2.8400
C2A···C6A^ii^	3.494 (2)	H31···H52	2.5900
C2A···O22^iv^	3.4158 (16)	H31···C11	2.8800
C3···O12	3.1653 (16)	H32···O22	2.6500
C3A···O22^iv^	3.1555 (16)	H32···H2^viii^	2.4300
C4A···O11^ii^	3.0969 (17)	H41···H4A^ix^	2.4500
C6A···O12^i^	3.4134 (17)	H41···C4A^ix^	3.0700
C6A···C2A^ii^	3.494 (2)	H42···H2	2.5000
C11···N1A	3.4331 (17)	H42···H61	2.5800
C11···O21	3.3436 (15)	H42···C21^viii^	3.0200
C21···O12	2.7964 (15)	H42···H1^vi^	2.5100
C21···O11^iii^	3.2513 (16)	H51···O21^vi^	2.9000
C1···H21^i^	2.886 (19)	H52···H31	2.5900
C2A···H5A^iii^	3.0000	H52···C11	2.8400
C4···H1^vi^	3.0000	H61···H2	2.5100
C4A···H41^vii^	3.0700	H61···H42	2.5800
C6···H21^i^	3.031 (19)	H62···O21^i^	2.8800
C11···H21A	2.774 (18)	H62···H21^i^	2.3700
C11···H1A	2.489 (15)	H62···O11	2.5100
			
C21—O21—H21	110.9 (11)	C2—C3—H31	110.00
C2A—N1A—C6A	122.30 (12)	C2—C3—H32	110.00
C6A—N1A—H1A	119.9 (9)	C5—C4—H41	109.00
C2A—N1A—H1A	117.6 (9)	C5—C4—H42	109.00
H21A—N21A—H22A	119.0 (14)	C3—C4—H42	109.00
C2A—N21A—H21A	121.9 (10)	C3—C4—H41	109.00
C2A—N21A—H22A	118.9 (11)	H41—C4—H42	108.00
C2—C1—C6	109.67 (10)	C6—C5—H51	109.00
C2—C1—C11	112.70 (10)	C4—C5—H52	109.00
C6—C1—C11	112.13 (10)	C6—C5—H52	109.00
C1—C2—C21	113.29 (10)	H51—C5—H52	108.00
C1—C2—C3	112.27 (10)	C4—C5—H51	109.00
C3—C2—C21	112.76 (11)	C5—C6—H62	109.00
C2—C3—C4	110.61 (11)	H61—C6—H62	108.00
C3—C4—C5	111.02 (12)	C1—C6—H61	109.00
C4—C5—C6	111.09 (12)	C1—C6—H62	109.00
C1—C6—C5	111.78 (11)	C5—C6—H61	109.00
O11—C11—C1	115.42 (11)	N1A—C2A—C3A	117.98 (12)
O11—C11—O12	123.26 (12)	N21A—C2A—C3A	124.10 (13)
O12—C11—C1	121.31 (11)	N1A—C2A—N21A	117.92 (12)
O21—C21—C2	113.41 (11)	C2A—C3A—C4A	119.42 (12)
O22—C21—C2	124.26 (12)	C3A—C4A—C5A	121.05 (13)
O21—C21—O22	122.23 (12)	C4A—C5A—C6A	118.21 (14)
C6—C1—H1	107.00	N1A—C6A—C5A	121.01 (14)
C2—C1—H1	107.00	C2A—C3A—H3A	120.00
C11—C1—H1	107.00	C4A—C3A—H3A	120.00
C1—C2—H2	106.00	C3A—C4A—H4A	119.00
C3—C2—H2	106.00	C5A—C4A—H4A	119.00
C21—C2—H2	106.00	C4A—C5A—H5A	121.00
C4—C3—H32	110.00	C6A—C5A—H5A	121.00
H31—C3—H32	108.00	N1A—C6A—H6A	120.00
C4—C3—H31	110.00	C5A—C6A—H6A	119.00
			
C6A—N1A—C2A—N21A	178.49 (13)	C3—C2—C21—O21	176.42 (10)
C6A—N1A—C2A—C3A	−1.86 (19)	C3—C2—C21—O22	−7.23 (17)
C2A—N1A—C6A—C5A	1.1 (2)	C1—C2—C21—O21	47.52 (14)
C6—C1—C2—C3	54.66 (13)	C1—C2—C21—O22	−136.14 (12)
C11—C1—C2—C21	58.14 (14)	C21—C2—C3—C4	174.78 (11)
C6—C1—C2—C21	−176.19 (10)	C2—C3—C4—C5	56.17 (14)
C2—C1—C11—O11	171.57 (10)	C3—C4—C5—C6	−56.90 (15)
C2—C1—C11—O12	−9.55 (16)	C4—C5—C6—C1	56.70 (14)
C11—C1—C2—C3	−71.01 (13)	N1A—C2A—C3A—C4A	0.9 (2)
C6—C1—C11—O12	−133.87 (12)	N21A—C2A—C3A—C4A	−179.53 (14)
C11—C1—C6—C5	71.16 (13)	C2A—C3A—C4A—C5A	0.9 (2)
C6—C1—C11—O11	47.24 (14)	C3A—C4A—C5A—C6A	−1.8 (2)
C2—C1—C6—C5	−54.84 (14)	C4A—C5A—C6A—N1A	0.8 (2)
C1—C2—C3—C4	−55.80 (14)		

Symmetry codes: (i) *x*, −*y*+3/2, *z*−1/2; (ii) −*x*+1, −*y*+1, −*z*+1; (iii) *x*, −*y*+3/2, *z*+1/2; (iv) −*x*+1, −*y*+1, −*z*+2; (v) −*x*, *y*+1/2, −*z*+3/2; (vi) −*x*, *y*−1/2, −*z*+3/2; (vii) −*x*+1, *y*+1/2, −*z*+3/2; (viii) −*x*, −*y*+1, −*z*+2; (ix) −*x*+1, *y*−1/2, −*z*+3/2.

### Hydrogen-bond geometry (Å, °)

**Table:**

*D*—H···*A*	*D*—H	H···*A*	*D*···*A*	*D*—H···*A*
N1A—H1A···O11	0.981 (15)	1.656 (15)	2.6223 (15)	167.7 (14)
N21A—H21A···O12	0.911 (18)	2.044 (17)	2.9361 (16)	166.3 (14)
N21A—H22A···O22^iv^	0.873 (16)	2.105 (16)	2.9103 (17)	153.1 (14)
O21—H21···O11^iii^	0.991 (19)	1.595 (19)	2.5806 (13)	172.9 (18)
C3A—H3A···O22^iv^	0.93	2.42	3.1555 (16)	136
C6A—H6A···O12^i^	0.93	2.55	3.4134 (17)	155
C6—H62···O11	0.97	2.51	2.8574 (16)	101

Symmetry codes: (iv) −*x*+1, −*y*+1, −*z*+2; (iii) *x*, −*y*+3/2, *z*+1/2; (i) *x*, −*y*+3/2, *z*−1/2.
